# Salicylic Acid Induces Changes in Mango Fruit that Affect Oviposition Behavior and Development of the Oriental Fruit Fly, *Bactrocera dorsalis*

**DOI:** 10.1371/journal.pone.0139124

**Published:** 2015-09-30

**Authors:** Kamala Jayanthi Pagadala Damodaram, Ravindra Mahadappa Aurade, Vivek Kempraj, Tapas Kumar Roy, Kodthalu Seetharamaiah Shivashankara, Abraham Verghese

**Affiliations:** 1 National Fellow Lab, Division of Entomology and Nematology, Indian Institute of Horticultural Research, Hessaraghatta Lake PO, Bangalore, Karnataka, India; 2 Division of Plant Physiology and Biochemistry, Indian Institute of Horticultural Research, Hessaraghatta Lake PO, Bangalore, Karnataka, India; University of Thessaly, GREECE

## Abstract

The Oriental fruit fly, *Bactrocera dorsalis* (Hendel) is an important quarantine pest around the globe. Although measures for its control are implemented worldwide through IPM and male annihilation, there is little effect on their population. Hence, there is a need for new strategies to control this minacious pest. A strategy that has received negligible attention is the induction of ‘natural plant defenses’ by phytohormones. In this study, we investigated the effect of salicylic acid (SA) treatment of mango fruit (cv. Totapuri) on oviposition and larval development of *B*. *dorsalis*. In oviposition choice assays, gravid females laid significantly less eggs in SA treated compared to untreated fruit. Headspace volatiles collected from SA treated fruit were less attractive to gravid females compared to volatiles from untreated fruit. GC-MS analysis of the headspace volatiles from SA treated and untreated fruit showed noticeable changes in their chemical compositions. Cis-ocimene and 3-carene (attractants to *B*. *dorsalis*) were reduced in the headspace volatiles of treated fruit. Further, reduced pupae formation and adult emergence was observed in treated fruit compared to control. Increased phenol and flavonoid content was recorded in treated fruit. We also observed differential expression of anti-oxidative enzymes namely catalase (CAT), polyphenoloxidase (PPO) and peroxidase (POD). In summary, the results indicate that SA treatment reduced oviposition, larval development and adult emergence of *B*. *dorsalis* and suggest a role of SA in enhancing mango tolerance to *B*. *dorsalis*.

## Introduction

Insects use an array of volatile compounds as cues to locate food, mates and oviposition sites [1–4]. Studies have demonstrated that insects use precise ratios of volatiles for host location [2,4]. Even subtle changes in volatile ratios of host plants confuse insects and alter their perception and orientation [5]. Plants have evolved with their insect pests and have developed an array of strategies for defense. One such strategy is the utilization of elicitors in priming and/or increasing or decreasing the production of certain volatile compounds upon insect attack [6]. Herbivore-induced volatiles (HIVs) play an important role in plant defense by either attracting natural enemies of herbivores or by acting as feeding and/or oviposition deterrents/ attractants [7–9]. Thus, exogenous applications of elicitors may impact insect-plant interaction through modified host plant volatile emissions.

Chemical elicitors viz., salicylic acid (SA), jasmonic acid (JA), ethylene, abscisic acid (ABA), gibberellic acid (GA_3_) are well studied and known to induce both direct and indirect defenses against insect pests [6–8,10–12]. Such induced responses in plants are important components of pest management and can be triggered by external application of elicitors [13]. Among the elicitors listed above, SA is well studied in non-woody plants for its role in regulating plant defense and in triggering ‘systemic acquired resistance’ (SAR) [14–19].

Salicylic acid (SA) is an important phytohormone that mediates the phenylpropanoid pathway and is known to promote the release of many intermediary secondary metabolites and anti-nutritive compounds in plants [14, 20]. It also modifies the characteristic plant volatile compositions [16–19] and enhances growth and vigor of plants [21, 22]. Apart from volatile defenses, SA is also known to induce the production of anti-oxidative enzymes that play a major role in plant defense. Among anti-oxidative enzymes, catalase (CAT), peroxidase (POD) and polyphenoloxidase (PPO) alter feeding behavior, growth and development of insect pests and play a major role in defending biotic and abiotic stress [23, 24]. Though many studies have explored the role of SA in inducing host plant defenses against pathogens, limited attempts have been made to study its role against insects. Exogenous treatment of SA to host plants, induced defense and modified the behavior of cotton bollworm, *Helicoverpa armigera* (Hübner) and the mite, *Tetranychus urticae* Koch [25,26]. Additionally, the effect of SA is well studied in herbaceous plants, but, studies on the effect of SA on woody plants are meager.

Mango (*Mangifera indica*) is a woody plant of high economic importance. Of several insect pests that infest mango, the fruit losses caused by the Oriental fruit fly, *Bactrocera dorsalis* (Hendel) (Diptera: Tephritidae) goes on unabated inspite of several management options [27–33]. Recently, emphasis is placed on the development of new preventative approaches that may minimize pesticide usage to achieve sustainable fruit fly management. Previous studies involving other tephritids such as the Caribbean fruit fly, *Anastrepha suspensa* (Loew) and Mediterranean fruit fly, *Ceratitis capitata* (Wiedemann) clearly showed that exogenous application of phytohormones (GA_3_) on fruit clearly deterred fruit flies and altered their oviposition behavior [34–39]. Further, GA_3_ application was found to offer viable means for reducing grapefruit susceptibility to *A*. *suspensa* in the field by reducing fruit attractiveness, ovipositional acceptability and physiological suitability for larval development by delaying peel senescence (37–39). However, use of host plant defense pathways that can be switched on or primed by exogenous application of phytohormones is not explored in mango. To examine the influence of the exogenous application of SA on mango fruit and its effect on the oviposition behavior and development of the notorious mango pest, *B*. *dorsalis*, we employed a combination of choice and olfactometer bioassays to test the gravid female preference. Further, we tested for biochemical changes between SA treated and untreated mango fruit.

## Materials and Methods

### Chemicals

Salicylic acid, hydrogen peroxide, catechol, guaiacol, polyvinylpyrrolidone, tris buffer, sodium phosphate, and di-sodium phosphate were purchased from Himedia, Bangalore, India. All chemicals used were of analytical grade (≥99% purity).

### Insects

The Oriental fruit fly, *B*. *dorsalis* from a laboratory colony was reared on banana (cv. Elakki) for six generations, with one generation cycle of one month and maintained at ambient conditions (27 ± 1°C, 75 ± 2% RH and 14L: 10D h photoperiod) in the Fruit Entomology Laboratory, Division of Entomology and Nematology, Indian Institute of Horticultural Research (IIHR), Bangalore, India [40]. Fruit were exposed to gravid females and placed on fine sterilized sand to allow pupation [40]. Pupae were separated by sieving the sand and placed in screened cages for adults’ emergence. Emerged flies were maintained in wooden nylon-screened holding cages (30×30×30 cm^2^) and provided with yeast, sugar and moistened cotton swabs *ad libitum*. Gravid females (15-days old) were used for all behavioral assays.

### Test fruit

Mature mango (cv. Totapuri, a susceptible variety to *B*. *dorsalis*) [41] fruit were collected from the experimental orchard of the Indian Institute of Horticultural Research, Bangalore. Healthy fruit without any scars at harvest were selected (randomly from two trees), rinsed with tap water and left to dry before use.

### SA preparation and application

Different concentrations of salicylic acid [0.5, 1.0, 1.5, 2.0 millimolar (mM)] were prepared in distilled hot water. A hand held atomizer (1 L capacity, Kisan Agri Sprayer, Mumbai, India) was used for uniform application of different doses on fruit. Fruit treated with distilled water were used as control. The fruit were sprayed in the laboratory with SA continuously for four days (one spray per day) and then used in bioassays.

### Oviposition preference assay

One salicylic acid treated along with one untreated mango fruit (*n* = 5 replicates per dose treatment) were exposed to gravid female *B*. *dorsalis* for oviposition to take place. Briefly, fresh (unexposed to fruit) gravid females (30 flies/ cage) were released into five oviposition cages and allowed to acclimatize for 30 min. In each treatment, the SA treated fruit along with a control mango fruit were placed into an oviposition cage (0.62m length x 0.62m wide x 0.62m height) and exposed to fruit flies for 12h. After the exposure period, number of oviposition punctures and the number of eggs laid per puncture were recorded under a stereomicroscope.

### Application of SA at different time intervals

Mangoes treated with SA (1 mL of 2 mM solution per fruit, n = 6) were exposed to gravid *B*. *dorsalis* for oviposition at different post SA treatment time intervals viz., 1, 2, 3 days after the spray. For each treatment, the SA treated fruit along with a control mango fruit (*n* = 6) were placed into oviposition cages (0.62m length x 0.62m wide x 0.62m height). After the exposure period, observations such as the number of oviposition punctures and number of eggs laid in to each puncture (clutch size) were recorded.

### Field bioassay

The study was conducted in a 25 years old mango cv. *Totapuri* orchard of IIHR, Bangalore (12° 58’N; 77°35’E). The area of the experimental plot was one hectare with a total number of 400 mango plants, at 5 m (between the plants) x 5 m (between the rows) spacing. Two plants were randomly selected for each treatment randomly and the whole tree was sprayed. SA solution (2000 mL of 2 mM) was sprayed twice i.e., when mango fruit were at pea stage (12^th^ February, 2014) and lime stage (12^th^ March, 2014) using a hand sprayer. The fruit were allowed to develop on the trees. Upon maturity, fruit were randomly collected (*n* = 8) and brought to the laboratory for oviposition assays. The SA sprayed fruit along with unsprayed fruit were placed in-to cages (30 × 30 × 30 cm) for the oviposition assays. Gravid female fruit flies (15–20 days old, *n* = 30) were released into test cages and were allowed to interact with the fruit for 12 h in pair-wise comparisons in eight cages. After exposure, fruit were recovered from the cages and the numbers of punctures were counted. The infested fruit were placed in plastic containers containing sterilized sand to allow pupation. After 10–15 days, the numbers of pupae recovered from each fruit were counted.

### Fruit volatiles collection

Headspace volatiles from SA treated and untreated mango fruit of green mature stage (cv. *Totapuri*; *n* = 4) were collected using a customized air entrainment system. Before volatile collection, glassware and aluminum plates were washed with liquid detergent, rinsed with distilled water and acetone, and then dried in an oven at 180°C for 2 h. Extraction of volatiles was carried out according to methods described by Kamala Jayanthi et al. [42]. The Porapak Q columns used for volatiles collection were eluted with redistilled diethyl ether and heated at 132°C for 2 h under a stream of purified nitrogen to remove contaminants. Fruit were placed individually inside cylindrical glass vessels (180 mm H, 100 mm dia), open at the bottom and closed with a collection port at the top and an inlet port on the side. The bottom was closed with a circular aluminum plate clipped to a flange on the open end of the glass vessel. Air, purified by passage through an activated charcoal filter, was pumped into the vessel through the inlet port (400 mL/min). Volatiles were collected on Porapak Q (50 mg, 60/80 mesh; Supelco, Sigma Aldrich, St Louis, USA) placed in a glass tube (5 mm dia) inserted into the collection ports on the top of the vessels. Further, pumps drew air (300 mL/min) through these tubes. All connections were made with PTFE tubing with brass ferrules and fittings (Swagelok, India) and sealed with PTFE tape. Volatiles were collected from fruit for 24 h and the Porapak Q columns were eluted with 750 μl of redistilled diethyl ether. Volatile samples were stored in a freezer (−20°C) until further use.

### Olfactometer bioassays with fruit head space volatiles

To study the behavioral responses of gravid *B*. *dorsalis* to headspace volatiles of SA treated and untreated mango fruit, behavioral assays were carried out using a circular Perspex four-arm olfactometer [120 mm diam, 42] placed inside a cage (0.62m length x 0.62m wide x 0.62m height) illuminated from above by diffused, uniform lighting using a fluorescent bulb (15W) and surrounded by black light proof walls to prevent influence of any external visual stimuli. The experiments were conducted at ambient room temperature (27 ± 1°C). Prior to experiment, all glassware was washed with liquid detergent, rinsed with acetone and distilled water and baked in an oven overnight at 180°C. Perspex components were washed with Teepol solution, rinsed with 80% ethanol solution and distilled water, and left to air-dry. The bottom of the apparatus was lined with filter paper (Whatman No 1, 12 cm dia) and air was drawn through the four arms towards the center at 350 mL min^-1^. A single gravid female *B*. *dorsalis* was introduced into the central chamber through a hole in the top of the olfactometer. Each fly was given 2 min to acclimatize in the olfactometer, after which the experiment was run for 15 min for each replicate.

Fruit fly behavioral responses were studied for headspace volatiles of SA treated and untreated mango fruit in the four-arm olfactometer (dual choice bioassay) as described by Kamala Jayanthi *et al*. [42]. Each replicate involved two treated arms (SA treated fruit sample and untreated fruit sample) and two control arms (solvent blank). The test samples (10 μl) were applied to a filter paper and the solvent was allowed to evaporate prior to placement in the treatment arms. The filter paper was then placed at the end of the treated side arm. Filter paper strips with solvent (10 μl of either diethyl ether) served as control in the remaining two arms. Ten (n = 10) replicates were carried out. Observations on the time spent and number of entries into each arm were recorded using Olfa software (F. Nazzi, Udine, Italy). The apparatus was rotated 90° every 2 min to eliminate any directional bias in the bioassay cage.

### GC-MS analysis

Sample of volatiles (1μl) was injected into the column inlet for gas chromatographic analysis and remained in the inlet for 10 min to be desorbed. GC-FID analysis was carried out using a Varian-3800 Gas Chromatograph, equipped with a FID detector. Nitrogen (1 mL/min) was used as the carrier gas. The components were separated on VF-5 capillary column, (Varian, USA) (30 m × 0.25 mm I.D. and 0.25 μm film thicknesses). The injector temperature was set at 260°C and all injections were made in split mode (1:5). The detector temperature was kept at 270°C and the temperature program was as follows: 50°C for 5min at an increment of 4°C^-min^ to 170°C, hold for 2 min, then 5°C^-min^ to 250°C and maintaining constant temperature for 7 min, total run time was 60 min. Quantification of volatiles was performed using a single point external standard quantification method using authentic samples of standards [43]. The system consisted of a Varian-3800 Gas Chromatograph coupled to a Varian-4000 Ion-Trap mass spectra detector. The ion trap, transfer line and ion source temperatures were maintained at 200°C, 240°C and 210°C respectively. A fused-silica capillary column VF-5MS (Factor four) (Varian, USA), with 30 m × 0.25 mm I.D., 0.25 mm film thickness was used for the analysis. Helium was used as carrier gas with the flow rate of 1 ml^-min^. The mass spectrometer was operated in the external electron ionization mode of 70 eV, with full mass scan-range of 45–450 amu. Temperature program for column was similar to that of the GC-FID analysis described above. Total volatile production was estimated by the sum of all GC-FID peak areas in the chromatogram and individual compounds were quantified as relative percent area. Individual volatile compounds were identified by comparing the retention index that was calculated by using homologous series of n-alkanes (C_5_ to C_32_ procured from Sigma-Aldrich) as standard [44] and comparing the MS spectra with spectral libraries (Wiley and NIST-2007).

### Effect of SA treatment on development of *B*. *dorsalis*

For this bioassay, SA treated and untreated fruit were placed in plastic containers with sterilized sand. The fruit peel was slightly cut open to facilitate introduction of larvae. First instar larvae of *B*. *dorsalis* (n = 100) obtained from a closed laboratory colony (maintained at 27 ± 1°C, 75 ± 2% RH and 14L: 10D h photoperiod) were placed in each fruit using fine camel-hair brush and allowed to settle. In this experiment, we used four fruit (*n* = 4) for each treatment. Each fruit was kept in a separate container. The containers were kept at ambient conditions 27 ± 1°C, 75 ± 2% RH and 14L: 10D h photoperiod for 10–15 days to allow larval development and pupae formation.

### Extraction and estimation of total phenol and flavonoids

The SA treated and untreated mango fruit (*n* = 3; 1g in 20 mL) were extracted with methanol (80%). The extract was concentrated to get a final volume of 10 mL. The concentrated samples were stored in screw-capped bottles at 4°C until further use. Total phenols present in methanol extracts were estimated by Folin-Ciocalteu method [45]. Methanol extracts were mixed with FCR reagent and the subsequent intensity of color development with 20% sodium carbonate reagent was measured at a wavelength of 700 nm using a UV-Vis spectrophotometer (Beckman DU64, Switzerland). Results were expressed as milligrams of gallic acid equivalents. Total flavonoids present in the samples were determined as described by Chun *et al*. [46]. Briefly, methanol extract was mixed with 0.3 mL of 5% NaNO_2_ followed by 0.3 mL of 10% AlCl_3_. After 1 min, 2 mL of 1M NaOH was added and diluted to 10 mL with double distilled water and mixed thoroughly. The absorbance was measured spectrophotometrically at 510 nm and expressed as catechin equivalents.

### Enzyme assay

Pulp of SA treated and untreated mango fruit (5g, n = 3) were homogenized in a pre-chilled pestle and mortar in 5 mL of ice-cold 50 mM Tris-HCl buffer (pH 7.4) containing a pinch of polyvinylpyrrolidone (PVP). The extract was centrifuged at 10000g for 10 min at 4°C. The supernatant was used as the enzyme source for the determination of activity. The protein concentration of the supernatant was determined using Lowry’s method [47] with bovine serum albumin as a standard.

We analyzed the activity of antioxidative enzymes viz., catalase, peroxidase, polyphenoloxidase in both SA treated and untreated fruit respectively (n = 3). Catalase activity [48] was determined with minute modifications by adding 0.1 mL of crude enzyme to 2.9 mL of 40mM H_2_O_2_ (dissolved with 50mM sodium phosphate buffer, pH 7.0) as a substrate. The hydrolysis of H_2_O_2_ was measured by the decrease in absorbance at 240 nm. One unit of catalase converts 1μmol of H_2_O_2_ per minute. Catalase activity was expressed in units per gram fresh weight (FW). Peroxidase (PO) activity [49] was analyzed by using guaiacol as substrate. The reaction mixture consisting of 0.5 mL of crude extract, 2 mL of guaiacol (100 mM sodium phosphate, pH 6.4 and 4 mM guaiacol) was incubated for 5 min at 30°C. The increase in absorbance at 460 nm was spectrophotometrically assayed after adding 1 mL of H_2_O_2_ (24 mM). Polyphenol oxidase (PPO) activity was estimated according to the method described as per Mayer and Hare [50]. The reaction mixture consisted of 2.9 mL of 0.1 M sodium phosphate buffer (pH 6.8), 0.1 mL of enzyme source and 0.1 mL of substrate (0.05 M catechol). Absorbance was read at 420 nm for 3 min at 30 sec interval. One unit of PPO was defined as the change in absorbance by 0.1 units per minute under conditions of the assay.

### Statistical analyses

Olfactometer bioassay data (time spent in each odour field, SA treated fruit vs. untreated fruit vs. solvent control) were compared by a non-parametric Kruskal-Wallis and Dunn’s multiple comparision test (α = 0.05). The data of post SA treatment time intervals were subjected to repeated measures (mixed model) two-way ANOVA with Bonferroni post test to compare means. Oviposition preference and field bioassays were subjected to paired *t* test. While, all other assays were compared using unpaired *t* test. All analyses were carried out using Graph Pad Prism software (Ver. 5.0.1) for Mac OS X.

## Results

### Effect of SA treatment on oviposition punctures and clutch size by *B*. *dorsalis*

**Fig 1:**
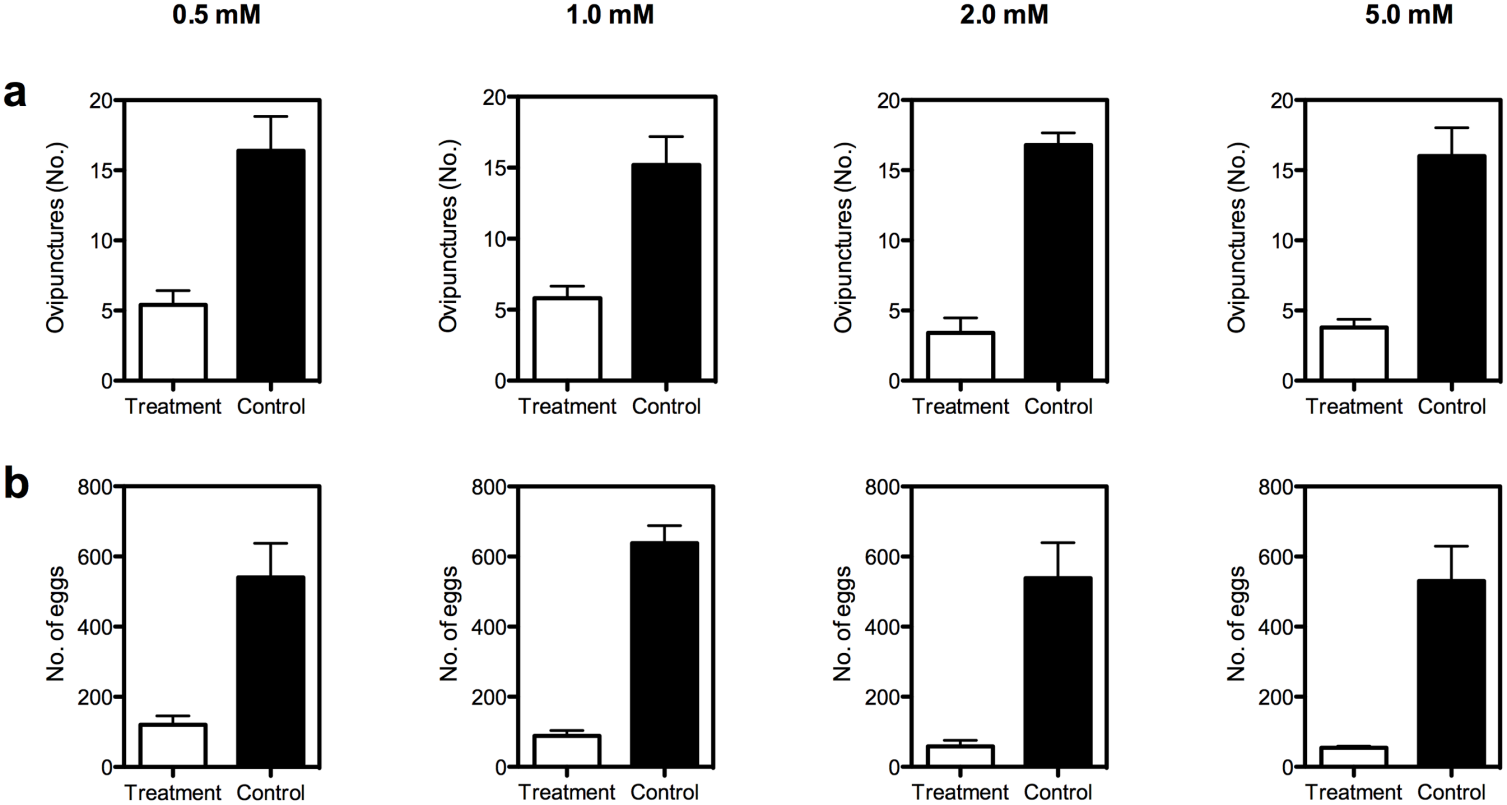
Effect of salicylic acid (SA) on the oviposition of *B*. *dorsalis*. **(a)** punctures (b) Eggs per puncture. Decreased number of punctures and eggs were recorded in SA treated fruit when compared to the control fruit. (paired *t* test, ovipunctures (0.5 mM: *t* = 7.59, *df* = 4, *P* = 0.002; 1.0 mM: *t* = 3.81, *df* = 4, *P* = 0.02; 2.0mM: *t* = 11.49, *df* = 4, *P* = 0.0003; 5mM: *t* = 6.89, *df* = 4, *P* = 0.002); eggs per puncture (0.5 mM: *t* = 5.15, *df* = 4, *P* = 0.01; 1.0 mM: *t* = 9.57, *df* = 4, *P* = 0.001; 2.0mM: *t* = 5.49, *df* = 4, *P* = 0.01; 5mM: *t* = 4.78, *df* = 4, *P* = 0.01). Error bars = Standard error of mean.

The number of punctures (Fig 1a) was significantly (0.5 mM: *t* = 7.59, *df* = 4, *P* = 0.002; 1.0 mM: *t* = 3.81, *df* = 4, *P* = 0.02; 2.0 mM: *t* = 11.49, *df* = 4, *P* = 0.0003; 5 mM: *t* = 6.89, *df* = 4, *P* = 0.002) different in SA treated fruits compared to their controls. The lowest number of punctures was recorded in 2.0 mM SA treatment followed by 5 mM and 0.5 mM SA treatments (Fig 1a). The untreated control fruit received the highest number of punctures. The number of eggs laid per fruit was also significantly (0.5 mM: *t* = 5.15, *df* = 4, *P* = 0.01; 1.0 mM: *t* = 9.57, *df* = 4, *P* = 0.001; 2.0 mM: *t* = 5.49, *df* = 4, *P* = 0.01; 5 mM: *t* = 4.78, *df* = 4, *P* = 0.01) lower in SA treated fruit compared to controls (*see* Fig 1b).

### Effect of post SA treatment time intervals on *B*. *dorsalis*

**Fig 2:**
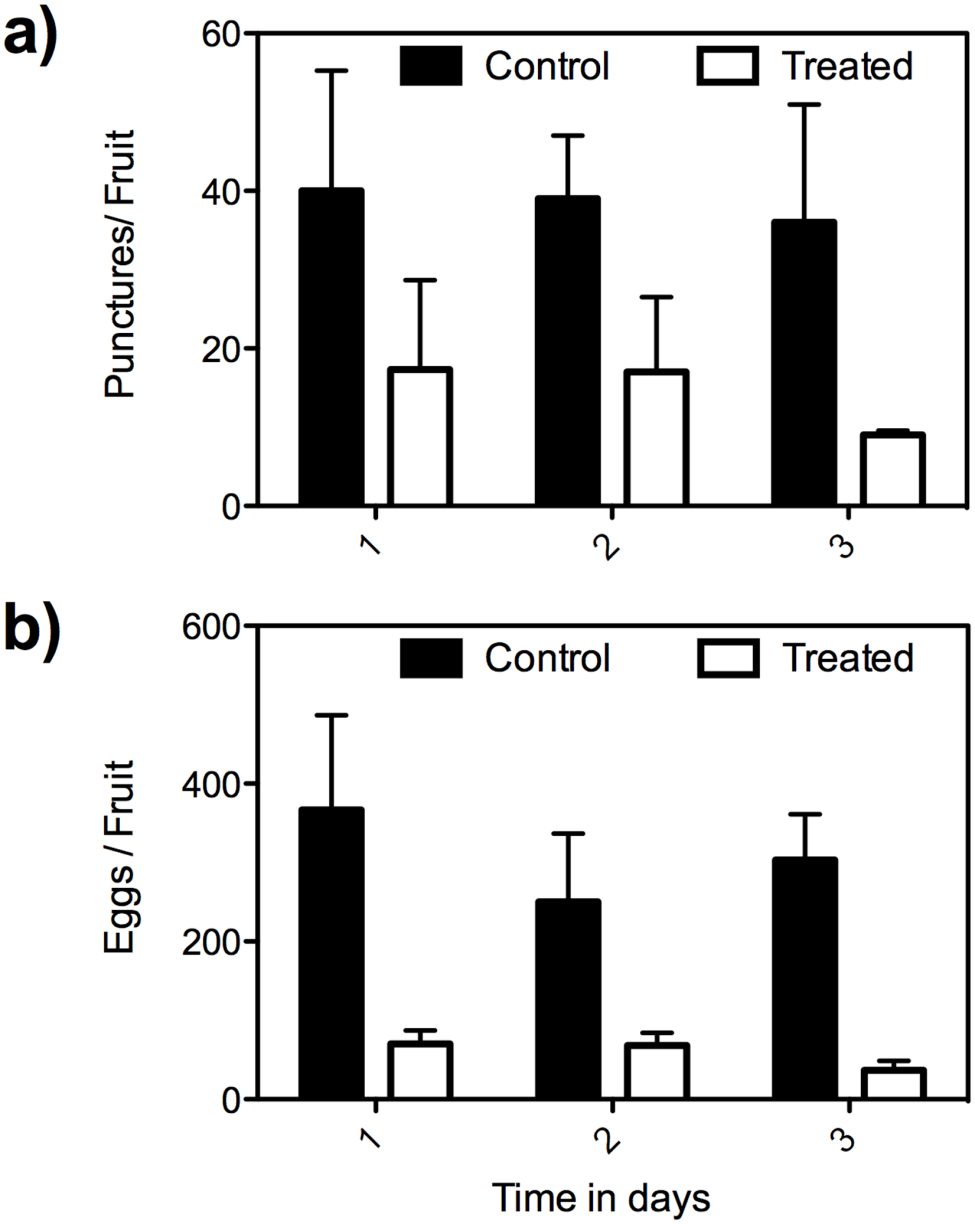
Effect of SA post treatment interval (days) on the oviposition of gravid female *B*. *dorsalis*. Repeated measures (mixed model) two-way ANOVA with Bonferroni post test (a) punctures (*F* = 6.86; *edf* = 20; *P* = 0.03) (b) Eggs per puncture (*F* = 130.20; *edf* = 20; *P* < 0.0001). Error bars = Standard error of mean.

A two-way repeated measures ANOVA indicated that the main effect of SA treatment was significant in terms of decreased oviposition punctures (*F* (2,20) = 47.60; *P* = <0.0001) and clutch size (*F* (2,20) = 45.81; *P* < 0.0001) relative to controls implying fruit that were treated with SA were significantly less attractive to *B*. *dorsalis*. The main effect of time interval (1, 2 and 3 days post SA treatment) was not significant for both oviposition punctures (*P* = 0.75) and eggs per puncture (*P* = 0.28) indicating all post SA treatments are equally effective. Post hoc analyses employing Bonferroni correction revealed that the numbers of oviposition punctures and eggs per puncture were significantly less in SA treated fruit irrespective of post SA treatment time intervals [1 day (oviposition punctures: *t* = 3.78, *P*<0.01; eggs per puncture: *t* = 4.67, *P* <0.001), 2 days (oviposition punctures: *t* = 3.67, *P* <0.01; eggs per puncture: *t* = 2.86, *P* <0.05), 3 days (oviposition punctures: *t* = 4.50, *P* <0.01; eggs per puncture: *t* = 4.20, *P* <0.01)] (Fig 2a and 2b).

### Field bioassay

**Fig 3:**
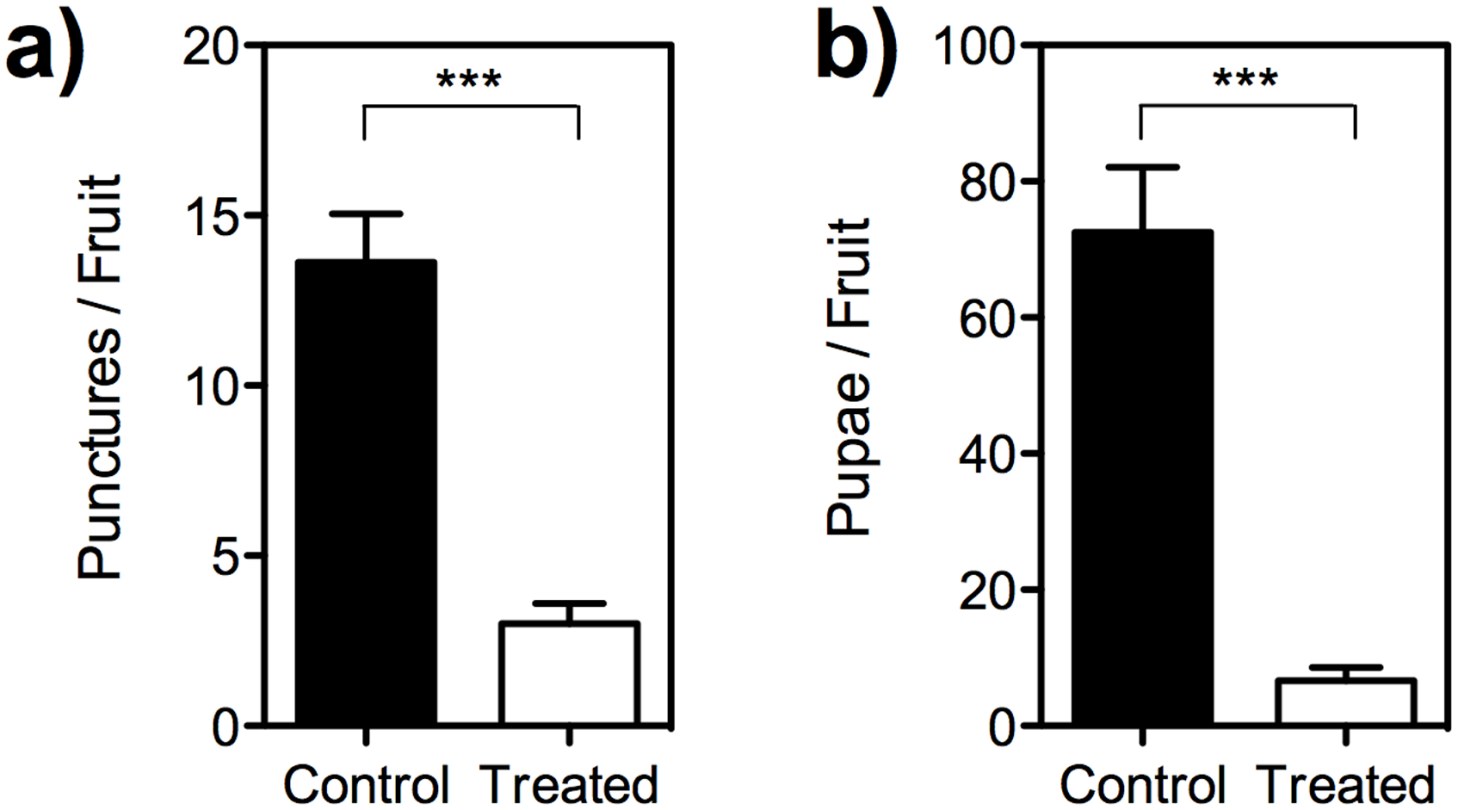
The response of female *B*. *dorsalis* to the SA treated fruit from the field. (a) Ovipunctures (b) the number of pupae emerged per fruit. The field experiment data shows that the number of punctures and pupae was decreased in the SA treated when compared to the control fruit (paired *t* test, *t* = 8.12, *df* = 7, *P* = 0.0001 [ovipunctures], *t* = 6.46, *df* = 7, *P* = 0.0003 [pupae]). Error bars = Standard error of mean.

There were significant differences in the number of punctures between SA treated and control fruit (*t* = 8.12, *df* = 7, *P* = 0.0001) (Fig 3a). Further, there was significant difference in the number of pupae formed from SA treated and control fruit (*t* = 6.46, *df* = 7, *P* = 0.0003) (Fig 3b).

### Olfactometer bioassay with headspace volatiles

**Fig 4:**
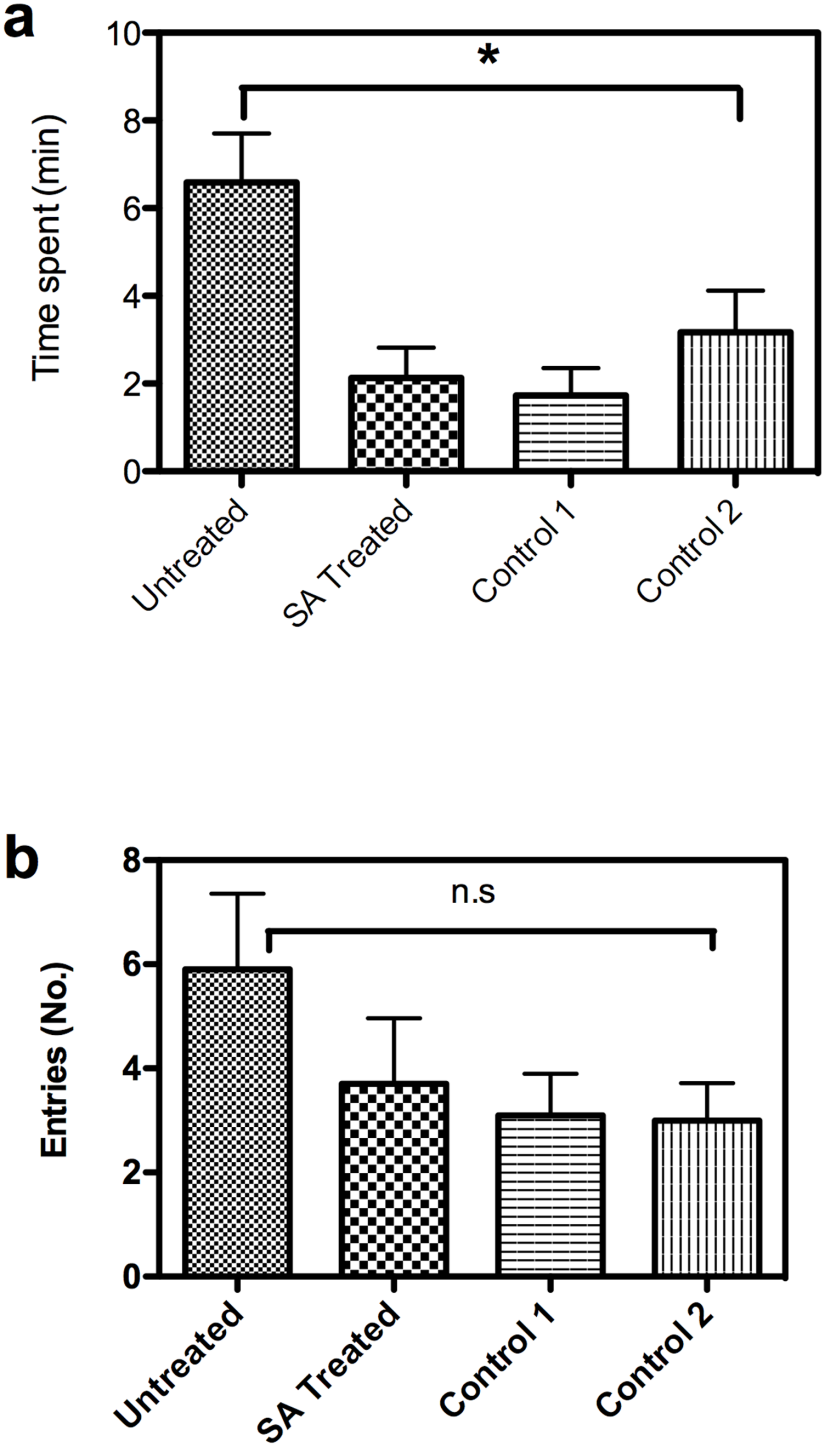
Behavioral response of female *B*. *dorsalis* to SA treated and untreated mango fruit (cv. Totapuri) volatiles in the olfactometer. (a) Time spent in SA treated, untreated and control arms (N = 10) (b) Number of Entries in SA treated, untreated and control arms (N = 10). The time spent and number of entries, significantly differed between untreated to treated fruit. (non-parametric Kruskal-Wallis and Dunn’s multiple comparison test, (*P* = 0.007) [time spent]; *P >* 0.05 [entries]). Error bars = Standard error of mean. *Significantly different from SA treated and control arms; n.s. = non-significant].

We assayed the behavior of gravid female *B*. *dorsalis* in four-arm olfactometer to headspace volatiles of both treated and control fruit. The time spent by gravid female *B*. *dorsalis* was significantly more in control arm (*P* = 0.007) than treated arm (Fig 4a). However, no significant difference was noticed between treated and control arms for number of entries made by gravid females (*P* > 0.05) (Fig 4b).

### GCMS analysis

**Fig 5:**
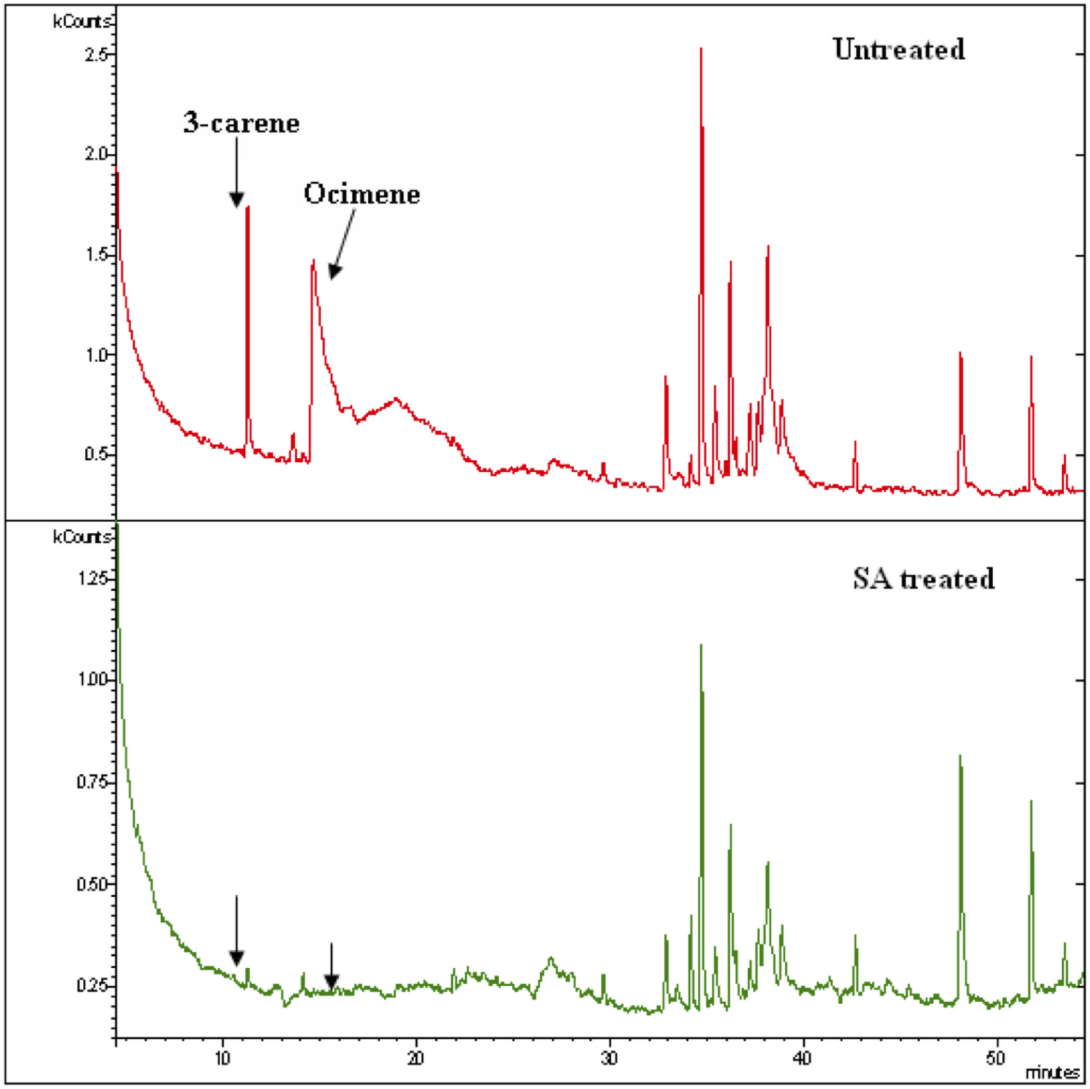
GCMS chromatogram of untreated and SA treated Totapuri fruit volatiles. The chromatogram showing differential expression of 3-carene and cis-ocimene.

The headspace volatiles subjected to GC-MS analysis revealed a clear difference in volatile composition between control and SA treated fruit. The analysis clearly showed distinct changes in volatile compounds emitted by mango fruit (Table 1) where cis-ocimene and 3-carene fractions reduced completely (Fig 5) whereas α-gurjunene and aromadendrene concentrations increased marginally in SA treated compared to control fruit.

**Table 1:** GCMS analysis of volatile compounds from untreated and SA treated mango fruit (Cv. Totapuri). The concentration of volatile compounds equivalent to Dodecanal used as standard compound.

	Untreated	SA treated
Compounds	(pg/mL)	(pg/mL)
α-Pinene	0.076	0.003
3-Carene	0.010	ND
cis-Ocimene	0.354	ND
α-Cubebene	0.045	0.013
α-Gurjunene	0.010	0.013
β-Caryophyllen	0.141	0.058
Azulene	0.037	0.010
α-Copaene	0.007	ND
Unknown	0.062	0.024
(+)-Aromadendrene	0.003	0.004
Guaiene	0.026	0.009
β-Selinene	0.009	0.002
β-Bulnesene	0.062	0.025
δ-Cadinene	0.038	0.005

ND = Not detected.

### Effect of SA treatment on larval development and adult emergence of *B*. *dorsalis*

**Fig 6:**
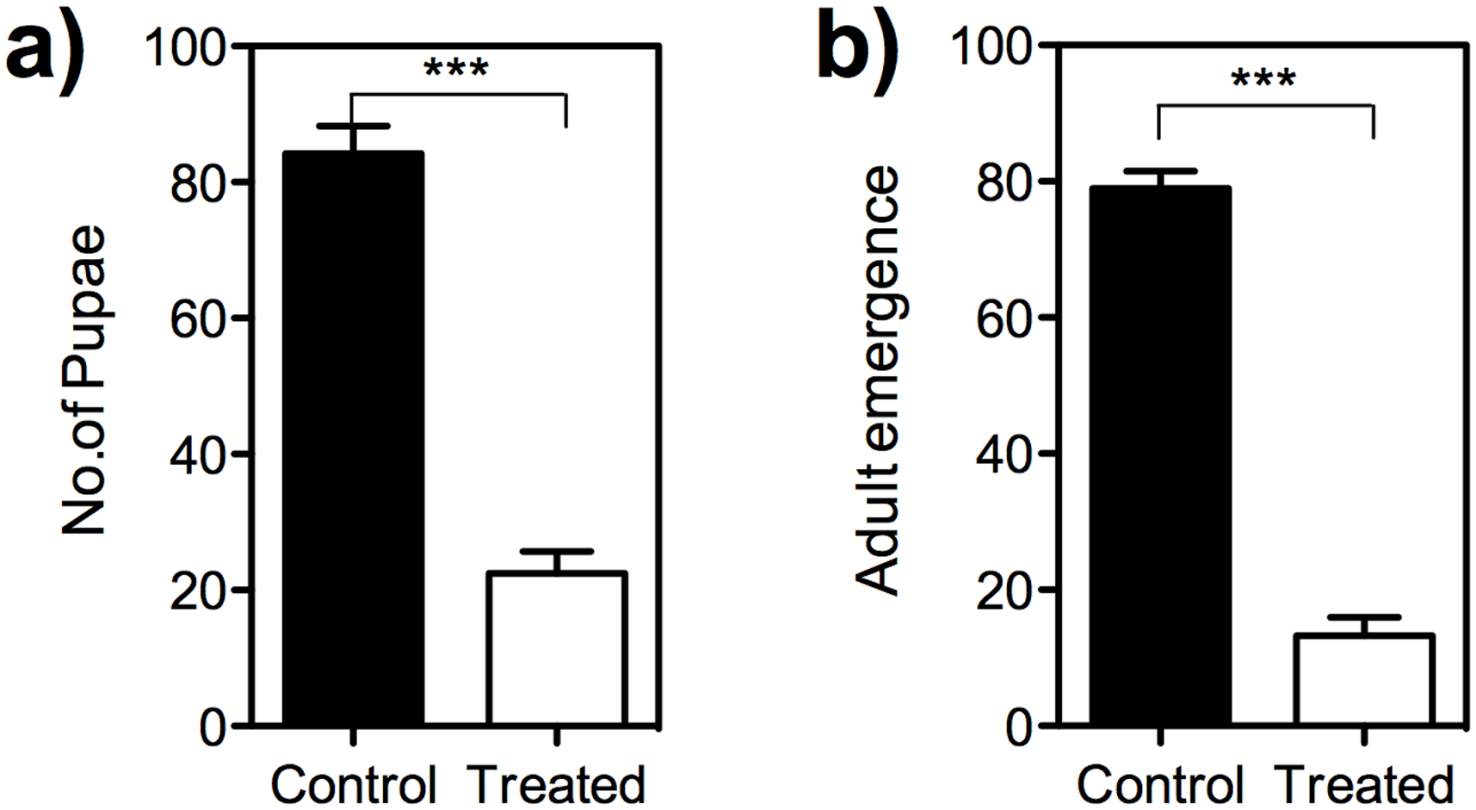
The assay of *B*. *dorsalis* development in the control and SA treated fruit. (a) The number of pupae formed; (b) The number of adults emerged. The pupal and adult emergence decreased in the SA treated fruit when compared to the control (unpaired *t* test, *t* = 11.93, *df* = 6, *P* = 0.0001 [pupae], *t* = 17.96, *df* = 6, *P* = 0.0001 [adults emerged]). Error bars = Standard error of mean.

In this assay, we allowed I instar larvae of *B*. *dorsalis* to develop and reach the pupal stage in SA treated and control fruit. The result showed that only 22.5% larvae reached the pupal stage in SA treated fruit as against 84.25% in the control. Further, from these pupae only 58.89% adult emergence was noticed in SA treated fruit compared to control which recorded 93.77% adult emergence. Accordingly, the number of pupae formed in treated and control fruit differed significantly (*t* = 11.93, *df* = 6, *P* = 0.0001) (Fig 6a). The number of adults emerged also showed a significant difference (*t* = 17.96, *df* = 6, *P* = 0.0001) between control and treated fruit (Fig 6b).

### Effect of SA on total phenol and flavonoids

**Fig 7:**
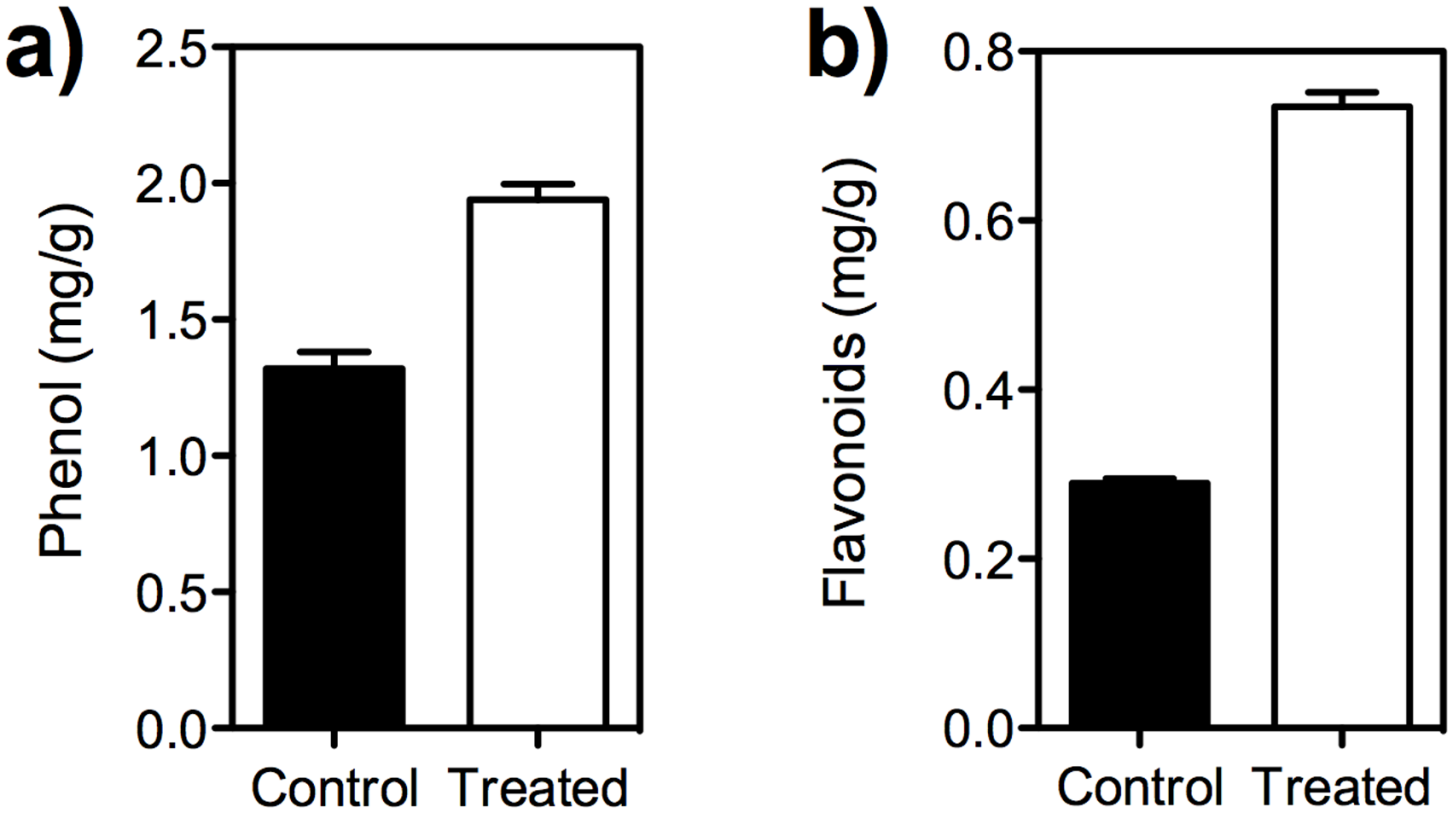
Effect of SA on the total phenol and flavonoids content. (a) Total phenols; (b) Total flavonoids. The total phenol and flavonoid levels were increased in the 2mM SA treated fruit when compared to the control fruit (unpaired *t* test, *t* = 7.38, *df* = 4, *P* = 0.0009 [phenols], *t* = 24.92, *df* = 4, *P* = 0.0001 [flavonoids]).

The total phenol content was significantly higher (*t* = 7.38, *df* = 4, *P* = 0.0009) in treated fruit when compared to the control fruit (Fig 7a). Similarly the total flavonoids content also differed significantly between treated (*t* = 24.92, *df* = 4, *P* = 0.0001) and untreated fruit (Fig 7b).

### Effect of SA on antioxidative enzymes

**Fig 8:**
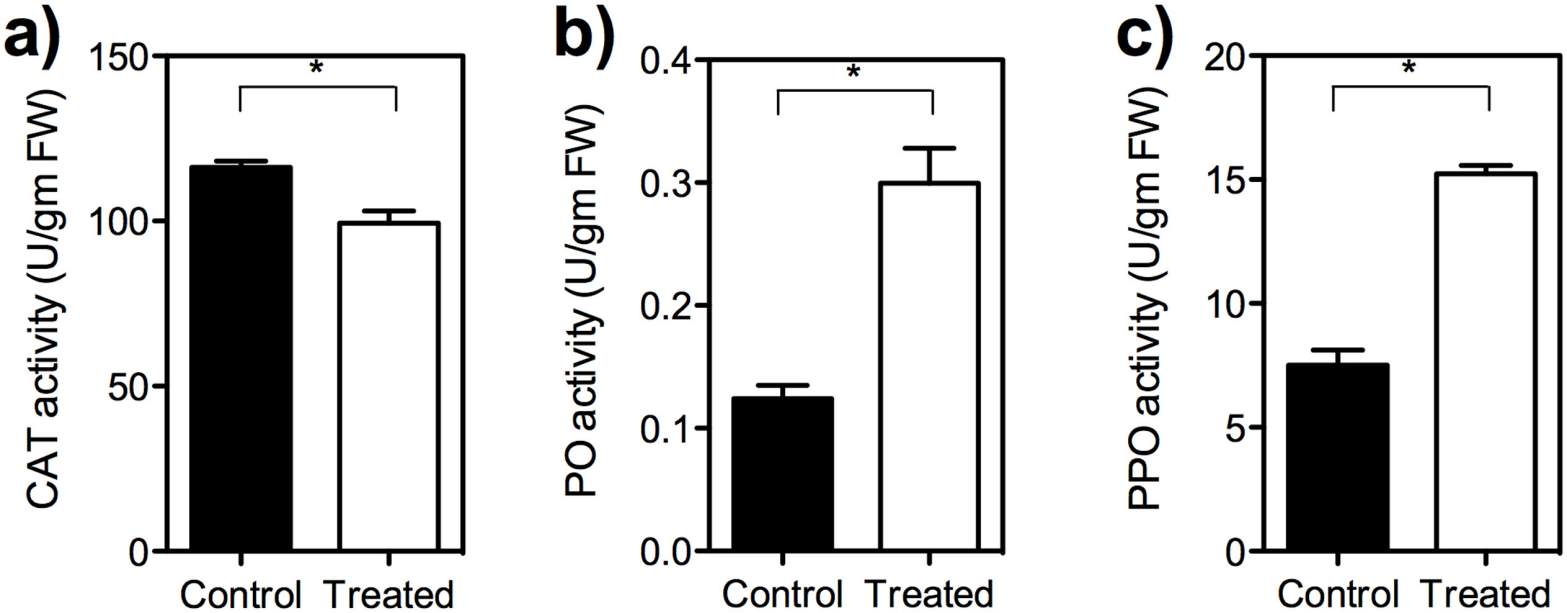
The effect of SA on the expression of antioxidative enzymes. (a) Catalase; (b) Peroxidase and (c) Polyphenoloxidase activity. The results showed inhibition of catalase activity and an increase of peroxidase and polyphenoloxidase activities in the treated fruit (unpaired *t* test, *t* = 4.01, *df* = 4, *P* = 0.01 [CAT]; *t* = 5.76, *df* = 4, *P* = 0.004 [POD]; *t* = 11.30, *df* = 4, *P* = 0.0002 [PPO]).

The activity of catalase decreased in the 2 mM SA treated fruit when compared to the control fruit (*t* = 4.01, *df* = 4, *P* = 0.01) (Fig 8a). Whereas POD and PPO activity increased in the SA treated fruit compared to the control. The activity of POD in the 2 mM SA-treated fruit was relatively higher than that in control fruit (*t* = 5.76, *df* = 4, *P* = 0.04) (Fig 8b). Similar trend was observed for PPO activity also (*t* = 11.30, *df* = 4, *P* = 0.0002) respectively (Fig 8c).

## Discussion

Finding a suitable host for oviposition is crucial to all phytophagous insects [51] and olfaction plays an important role in enabling the host plants recognition [52, 53]. Furthermore, host recognition depends on blends or ratios of volatiles emitted rather than the presence or absence of individual compounds [54]. This is the first time we show that the SA induced changes in mango fruit affects the attraction of female Oriental fruit fly, *B*. *dorsalis*. To identify a suitable oviposition site, insects mainly depend on host released volatile blends [2], particularly, during oviposition site selection [55]. Thus, females readily discriminate between oviposition sites of different quality to maximize larval survival and avoid competition from conspecifics for resources [4, 56–58]. Our results showed reduction in oviposition by *B*. *dorsalis* in SA treated fruit as the number of punctures and eggs were significantly reduced compared to untreated fruit. The reduction in oviposition as evidenced by decreased punctures and eggs reached peak by 3^rd^ day post SA treatment. Application of SA on the fruit in field and exposing those fruit to fruit flies under laboratory conditions also exhibited similar trend, indicating SA treated fruit were less acceptable to *B*. *dorsalis*. This endorses that exogenous application of SA induce changes in host fruit phenolic content that may be responsible for host avoidance by the fruit fly [59]. It is well recognized that salicylic acid potentially generates a wide array of metabolic responses in plants at extremely low concentrations (μM to mM) and these multifaceted responses can make the host unsuitable against herbivores through modulating host plant volatile emissions and chemical content thereby herbivore host finding and selection [59,60].

Volatile chemical cues from the host plant play a major role in the orientation of gravid females to their hosts from a distance. Thus perception of right mix of these volatile blends plays a pivotal role in host recognition and determines the probability of phytophagous insect alighting on a given host [54]. Our olfactometer results show that volatiles collected from untreated fruit attracted *B*. *dorsalis* whereas volatiles from SA treated fruit did not attract the flies. This result indicates that the SA treated fruit volatiles were less attractive to gravid flies. GC/MS analysis confirmed the complete absence of volatiles viz., cis-ocimene and 3-carene in SA treated fruit. These are reported to elicit significant EAG response as well as positive behavioural responses in gravid female *B*. *dorsalis* [42]. Therefore, these two chemical cues, cis-ocimene and 3-carene are important attractants and involved in host location of *B*. *dorsalis* [42]. The subsequent inhibition of cis-ocimene, 3-carene after the exogenous application of SA would have led to the observed altered behavior of *B*. *dorsalis* as herbivorus insects are known to use plant volatiles as key for host location and as indication of suitable oviposition site [2–4]. Similarly, reduced oviposition by *H*. *armigera* was noticed in groundnut cultivars after jasmonic acid/ salicylic acid application [17].

Further studies on effect of SA on larval development and adult emergence of *B*. *dorsalis* revealed significant reduction in pupae formation and adult emergence when larvae were reared on SA treated fruit (Fig 3b). The SA treated fruit showed high level of phenol and flavonoids content compared to control. Earlier studies report that salicylic acid treatment increased coumarins, phenolic acids, flavonoids and lignin concentration in plants [61–63]. Phenolic content in the host fruit affects the fruit fly development [64, 65]. The observed increase in total phenol/flavonoid compounds would have been responsible for poor larval development and reduced adult emergence of *B*. *dorsalis* [66]. Most of the flavonoids are growth inhibitors and cause abnormal development, growth inhibition and larval mortality [66]. An increase in phenols, flavonoids, lignin and hydroxyproline-rich glycoproteins was noticed in mangoes subsequent to post-harvest treatment by chemical elicitor, benzothiadiazole [67]. Application of salicylic acid resulted in satisfactory mite control in *Phaseolus vulgaris* and enhanced yields [68].

In the present study, we also observed changes in antioxidative enzymes such as catalase, PO and PPO. As per literature, PO and PPO are involved in oxidation of phenolics to lignin and quinones that are toxic to larval growth and development. In the present study, the decline in catalase activity and its associated reactive oxygen species (ROS) generation would have been further affected the *B*. *dorsalis* larval growth in SA treated fruit endorsing previous studies that report post SA treatment enhancement in antioxidative defense systems [69], and the inhibition of catalase activity in tobacco plant, and cherry fruit [70,71]. Induction of defense related enzymes (PAL, PPO, catalase, peroxidase, superoxide dismutase etc) and subsequent induced resistance to *Alternaria brassicae* and *Ralstonia solanacearum* was noticed following chemical elicitors application viz., SA, benzothiadozole in *Brassica juncea* and *Solanum melongena* respectively [72]. Similarly, the salicylic acid treatment resulted in increased endogenous H_2_O_2_ level that involved in resistance against *H*. *armigera* [73].

The Oriental fruit fly, *B*. *dorsalis* is a major pest on mango causing huge losses to farmers all over world and the pre-harvest management of fruit flies involves several strategies viz., male annihilation, methyl eugenol traps, proteinaceous food baits, and insecticidal cover sprays to minimize the losses at farm level [74]. Nevertheless, utilization of direct and indirect host-plant defense mechanisms and exploiting these plant signals for sustainable IPM is an area that yet to be explored in several perennial fruit crops. Our study clearly indicates that the exogenous application of SA on to mango fruit resulted in reduced oviposition by Oriental fruit fly, *B*. *dorsalis*. Our results showed that the host fruit compounds viz., cis-ocimene and 3-carene were sharply declined after SA treatment indicating that the absence of these volatiles along with associated biochemical changes would have been responsible for altered ovipositional behaviour of *B*. *dorsalis*. Further, reductions in the larval growth/ adult emergence were also found in SA treated fruit that can be attributed to an increase in the total flavonoids/ phenols and antioxidative enzymes viz., peroxidase and polyphenoloxidase. Enhanced levels of these plant defense compounds would have affected larval growth and development resulting in poor pupation and adult emergence of *B*. *dorsalis*. Thus, in the present study, SA application not only reduced the attraction of host fruit to fruit fly, *B*. *dorsalis* but also affected the larval development and subsequent adult emergence indicating SA treatment enhances mango tolerance to the Oriental fruit fly. There is a need for in depth studies on possibility of exogenous application of SA for fruit fly management in mango at field level and to understand the role of this chemical elicitor in strengthening our current pest management programs.
